# Seedless and Surfactant-Free Synthesis of Polyhedron Gold Nanocrystals Enclosed by High-Index Facets for Enhanced Electrochemical Detection of Benzoyl Peroxide in Flour

**DOI:** 10.3390/molecules29235691

**Published:** 2024-12-02

**Authors:** Zixuan Wang, Qianlong Gao, Kai Yao, Wei Ran, Ying Li, Yushen Jin, Bing Shao, Jiefang Sun

**Affiliations:** 1 School of Public Health, Capital Medical University, Beijing 100054, China; 112023010141@mail.ccmu.cn (Z.W.); jinyushen2010@126.com (Y.J.); shaobingch@sina.com (B.S.); 2 School of Chinese Medicine, Yunnan University of Traditional Chinese Medicine, Kunming 650500, China; gql1922383403@163.com (Q.G.);; 3 Beijing Center for Disease Prevention and Control, Beijing 100013, China; 4 State Key Laboratory of Environmental Chemistry and Ecotoxicology, Research Center for Eco-Environmental Sciences, Chinese Academy of Sciences, Beijing 100084, China; ranwei@163.com

**Keywords:** polyhedron gold nanocrystals, high-index facets, electronic oxidation, benzoyl peroxide sensing, protonated hydrazine

## Abstract

Polyhedron gold nanocrystals enclosed by high-index facets (HIF-Au NCs) are in high demand but are very difficult to prepare. To address this issue, we presented a simple, seedless method for synthesizing uniform HIF-Au NCs in an aqueous solution, which remarkably reduced the synthesis difficulty. Interestingly, the protonated N_2_H_4_ which served as both the reducing and capping agent played a crucial role in modulating the kinetic growth of the HIF-Au NCs. The resulting HIF-Au NCs exhibited distinct electronic oxidation inertness toward alcohol but demonstrated exceptional activity in the electrocatalytic oxidation of peroxides. To demonstrate their sensing capabilities, an electrode decorated with HIF-Au NCs was used to selectively detect benzoyl peroxide (BPO) in flour. BPO is a prohibited whitening agent that may be illegally added to flour and other products, posing potential health risks. The results demonstrate that this assay offers a promising method for the sensitive and selective detection of BPO. In conclusion, this research provides a straightforward pathway for obtaining HIF-Au NCs and further demonstrates their use in electronic sensing. It is expected that HIF-Au NCs will serve as a powerful tool in plasmon-enhanced spectroscopies, catalysis, and sensing applications.

## 1. Introduction

Electrochemical analysis is widely utilized in environmental detection [1], food safety [2], daily care [3,4], and medical diagnosis [5] due to its high sensitivity and selectivity, low cost, and ease of operation. Nanostructured metals, especially precious metals (Pt, Pd, Ru, Rh, Au, et al.), have been widely designed as electrocatalysts owing to their near-zero overpotential and excellent stability [6,7,8]. In this context, engineering the morphology and electronic structure offers opportunities for the synthesis of high-performance electrochemical sensors. Although they exhibit prominent sensing abilities owing to their distinctive properties which originate from structural anisotropy and rich surface chemistry [9,10], platinum group metal (Pt, Pd, Rh)-based electrochemical sensors are susceptible to inactivation by alcohol [11]. This issue restricts their sensing applications as many low-polar targets are extracted using alcohol prior to testing [12,13].

By contrast, gold nanocrystals (Au NCs), which show better resistance to catalyst poisoning, represent a good choice for electrochemical sensors [14,15]. Controlling the synthesis of Au NCs is an attractive direction for catalyst engineering and performance optimization in electro-sensors [16,17]. Constructing high-index-facet Au NCs (HIF-Au NCs) featuring well-defined sharp tips and edges with a high content of active low-coordination-number atoms has received particular interest in catalysis and sensing fields because they are more energetically and kinetically favorable than their low-index-facet counterparts [18,19,20,21,22]. Despite their outstanding properties, achieving a high quantity of HIF-Au NCs remains a challenge because their inherent thermodynamic instability leads to rapid atom deposition and facet loss during crystal growth [23]. To date, many efforts have been made to synthesis Au NCs with controlled sizes, morphologies, and crystalline phases to achieve the desired electro-sensing performance. These methods generally require high concentrations of surfactants [24,25] and gold seeds to modulate the exposed high-index facets. While these approaches yield Au NCs with high uniformity and shape control, the capping surfactants are difficult to remove, which inevitably blocks their catalytic interfaces. To address this issue, Sun et al. developed a series of electrochemical synthesis methods to obtain HIF-Au NCs in deep eutectic solvents (DESs) without using a surfactant. These methods have been successfully extended to the electrochemical synthesis of other metal NCs. However, the harsh conditions and technical complexity limit their widespread use.

Herein, we demonstrate a facile one-pot aqueous reduction method to engineer uniform and monodisperse HIF-Au NCs which only need a gold precursor and acidifying hydrazine hydrate (N_2_H_4_). The resulting HIF-Au NCs exhibited attractive features, including high surface-enhanced Raman scattering (SERS) activity and excellent electronic oxidation activity. Both high-sensitivity X-ray photoelectron spectroscopy (XPS) and Raman spectroscopy revealed that protonated N_2_H_4_ (at pH 1.5–4.0) provided moderate control of the formation of HIF-Au NCs through its adsorption on the surface of the synthesized nanocrystals. Notably, this method did not involve any surfactants, thereby preventing contamination of the electrochemically active sites. The HIF-Au NCs demonstrated enhanced sensitivity and specificity in the electro-oxidation reaction of peroxide. Furthermore, we successfully proposed a highly sensitive electrochemical method for detecting the highly toxic benzoyl peroxide (BPO) in flour. BPO, an illegal additive, may still be added to flour and other products to enhance color and luster, thereby compromising food nutrients and posing health risks.

## 2. Results and Discussion

### 2.1. Synthesis and Characterization of the HIF-Au NCs

**Figure 1:**
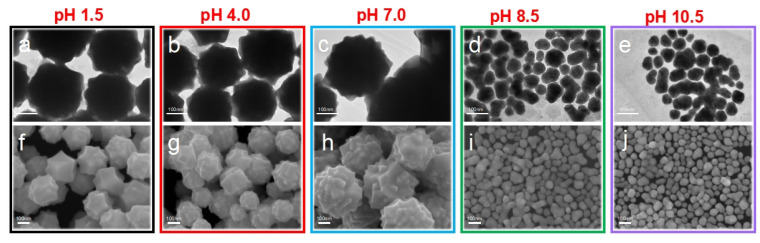
TEM (**a**–**e**) and SEM (**f**–**j**) images of the HIF-Au NCs prepared using N_2_H_4_ reducing agents of different pH values.

N_2_H_4_ is a strong alkaline reducing agent widely used in chemical engineering. However, it provides weak control over the atomic arrangement of metal surfaces. In this research, we found that N_2_H_4_ played a fundamental role in modulating the morphology and exposing facets of the synthesized Au NCs. The HIF-Au NCs were prepared using a one-step seedless wet-chemical aqueous synthesis. The N_2_H_4_, with a pH range from 1.5 to 10.5, served both as the shape-directing agent and reducing agent. As shown in the TEM and SEM images (Figure 1a–j), the Au NCs reduced from the acidified N_2_H_4_ (pH 1.5) exhibited rough, sharp angular surfaces with an average size of 260 nm (Au NC_pH1.5_). When the pH of the N_2_H_4_ solution was increased to 4.0, the particle sizes of the Au NCs (Au NC_pH4.0_) were maintained, but the dispersion of particle sizes became slightly larger than that of Au NC_pH1.5_. This result was consistent with the optical characteristics. 

**Figure 2:**
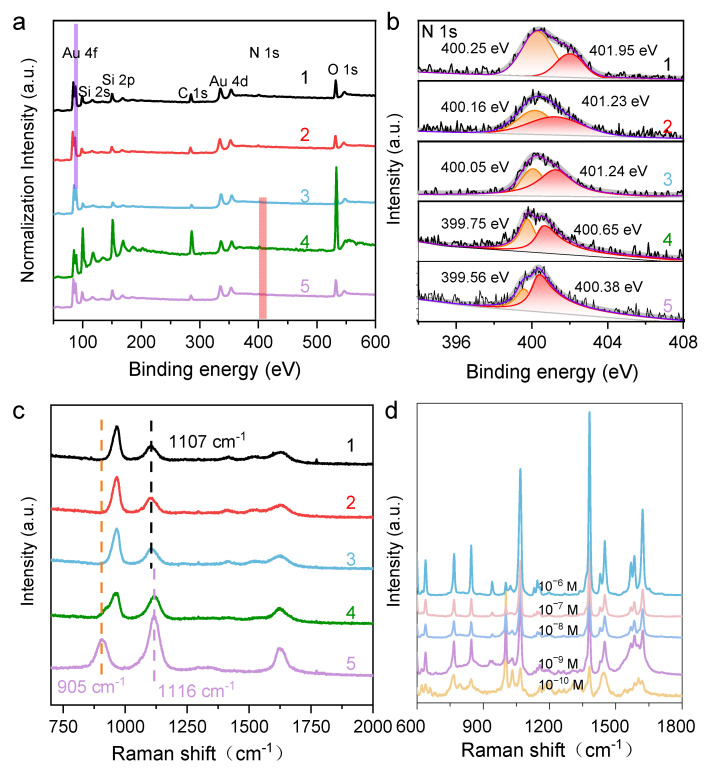
(**a**) HRXPS of the HIF-Au NCs synthesized using different protonated N_2_H_4_ solutions (pH from 1.5 to 10.5); (**b**) Splitting N1s peak of HRXPS; (**c**) Raman spectra of different protonated N_2_H_4_ solutions (pH from 1.5 to 10.5); lines 1, 2, 3, 4, and 5 represent the Au NC_pH1.5_, Au NC_pH4.0_, Au NC_pH7.0_, Au NC_pH8.5_, and Au NC_pH10.5_, respectively; (**d**) SERS enhancement of 2-MN using the HIF-Au NCs_pH1.5_ synthesized using this method.

As suggested from the results above, the protonated N_2_H_4_ played a fundamental role in modulating the growth of the Au NCs, ultimately determining their final structures. The proposed growth mechanism of Au NCs with {311} facets was supported by the XPS results, particularly the N1s XPS spectra of residual N species on the Au surface (Figure 2a,b). The spectrum at 400.6 eV is attributable to =NH^+^ species, indicating that N_2_H_4_ is chemically bound to the Au surface. A shoulder peak corresponding to -NH- was also observed at 399.6 eV, likely stemming from reacted N_2_H_4_ species. As pH decreases, the binding energy (BE) gradually increases. However, detailed curve fitting revealed that the main component remains at 400.7 eV, indicating that the =NH^+^ group is the dominant surface adsorbent. A new shoulder at 402.3 eV had a much higher BE, corresponding to -NH_2_ species. Overall, these results reveal the changes in N species under different pH conditions and demonstrate that protonated N_2_H_4_ plays a key role in modulating the size and exposing facets of the synthesized Au NCs. To explore the growth mechanism of the HIF-Au NCs, in situ Raman spectroscopy was used to inspect the changes in N_2_H_4_ under different pH conditions. As shown in Figure 2c, the original N_2_H_4_ aqueous solution (0.1 M, pH 10.5) featured strong bands at 905, 1116, and 1630 cm^−1^ in its Raman spectra, corresponding to the ρ(NH_2_), ν(N-N), and δ(NH_2_) vibration modes of N_2_H_4_, respectively. With the decrease in pH, the relative intensities of ρ(NH_2_) and δ(NH_2_) dropped significantly, while a new Raman band appeared at approximately 965 cm^−1^, which was consistent with the Raman spectral features of -NH_2_. At pH 7.0, the ρ(NH_2_) band of N_2_H_4_ disappeared, and the Raman band shifted from 1116 to 1107 cm^−1^. Notably, even at pH 1.5, the Raman band associated with ν(N-N) still exhibited moderate intensity, and no Raman bands attributable to NH_4_^+^ were observed. These results indicate that N_2_H_4_ gradually protonated into N_2_H_5_^+^ and ultimately N_2_H_6_^2^^+^, while its chemical structure remained stable with no chemical bond breaking occurring as the pH decreased.

Mechanically, the size and morphology of the Au NCs are determined by the nucleation and growth steps. Nucleation is influenced by the reduction rate of AuCl_4_^−^, while nanocrystal growth is determined by the adsorption strength of coexisting ions. Under basic conditions (pH 8.5–10.5), N_2_H_4_ demonstrated strong reducing ability but weak capping capacity, leading to explosive nucleation and an irregular morphology of the resulting Au NCs. Under neutral conditions (pH 7.0), the synthesized Au NCs showed polycrystals larger than those formed under acidic conditions. This was because the weaker electronic repulsion among newly growing Au seeds hindered their further growth into large NCs but allowed them to aggregate into larger nanostructures via an oriented attachment (OA) process. This assumption was further supported by the presence of rich defect sites, such as stacking faults and twin boundaries, as shown in the HRTEM images. Under acidic conditions (pH 1.5–4.0), with protonated N_2_H_4_, both nucleation and adsorption capacities were weakened. The slow reduction of AuCl_4_^−^ by N_2_H_5_^+^/N_2_H_6_^+^ slowed down Au nucleation, leading the newly formed Au(0) atoms to preferentially adsorb on the surfaces of existing Au NCs or to adopt a heterogeneous nucleation mechanism. Due to the weak adsorption of protonated N_2_H_4_ species, only high-index facets like {311} were blocked, allowing newly reduced Au atoms to deposit onto other facets. This explains the formation of large Au NCs exposing the {311} facet. 

**Figure 3:**
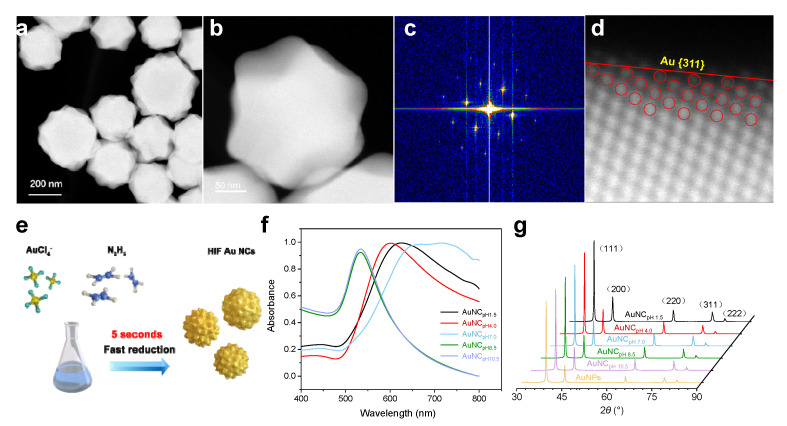
(**a**) HAADF-STEM image of the HIF-Au NC_pH1.5_ and (**b**) its zoomed-in image; (**c**) SAED pattern obtained from a Au NC_pH1.5_; (**d**) Cs-HAADF-STEM image of the {311} facet of a Au NC and its corresponding geometric model; (**e**) Schematic drawing of the synthesis of the Au NCs; (**f**) Normalized extinction spectrum and (**g**) XRD patterns of the Au NCs synthesized using different protonated N_2_H_4_ reducing agents (the major peaks are labeled using different colors to indicate the abundance of the respective facets).

Numerous small bulges were observed on the surface of the Au NCs, increasing their sizes to over 300 nm. These image results were in line with their extinction spectra (Au NC_pH7.0_), which displayed a distinct red shift and broadening. However, when basic N_2_H_4_ solutions at pH 8.5 or 10.5 were used as the reducing agents, only irregular spheroidal Au NCs of 40–60 nm in diameter with aspect ratios of 2–3 were obtained. The surfaces of these Au NCs were relatively smooth, lacking any angular features. Meanwhile, the longitudinal surface plasmon resonance (SPR) bands of these Au NCs (Au NC_pH8.5_, Au NC_pH10.5_) underwent a significant blueshift due to a decrease in the aspect ratio, with absorption peaks around 530 nm. HAADF-STEM further revealed that the arrangement of the polygonal surfaces was uniform, forming large concave corners of 126 and 143°, which are typically associated with HIF-Au NCs exposing the {311} facet (Figure 3a,b). The corresponding selected-area electron diffraction (SAED) pattern illustrates their single-crystal structure (Figure 3c). To verify their fine nanostructures, atomic-resolution spherical-aberration-corrected high-angle annular dark-field scanning transmission electron microscopy (Cs-HAADF-STEM, Figure 3d) was employed to observe the atomic arrangement of surface gold atoms. Based on the microfacet notation, Au {311} facets can be expressed as Au(s) − [2{100} × {111}], suggesting a stepped surface composed of terraces with two atomic columns of {100} symmetry separated by one atomic step of {111} symmetry. As shown in Figure 3f, the extinction spectra of Au NC_pH1.5_ and Au NC_pH4.0_ exhibited similar plasmonic bands, with the SPR peaks shifting slightly away from 630 and 600 nm, respectively. When using a neutral N_2_H_4_ aqueous solution (pH 7.0) as the reducing agent, the prepared Au NCs (Au NC_pH7.0_) retained a polyhedral nanostructure but showed significant increases in diameter and size distribution. To verify the crystalline nature of the Au NCs synthesized with different protonated N_2_H_4_, X-ray diffraction (XRD) patterns were used to characterize various crystal facets (Figure 3g). Peaks related to high-index facets, such as {311} and {220}, are clearly evident in the XRD pattern of the Au NCs synthesized with protonated N_2_H_4_ (pH 1.5 to 7.0). Notably, compared to typical spherical Au NPs synthesized by citrate reduction (80 nm), the ratio of I_{311}_ to I_{111}_ showed consistent enhancement as the pH of N_2_H_4_ decreased, confirming the polycrystalline nature of the Au NCs. The rough surface of the Au NCs significantly increased the surface area, thereby accelerating surface reactions such as the electrochemical oxidation of target molecules.

HIF-Au NCs have been reported to exhibit excellent SERS activities due to their well-defined tips, edges, steps, and kinks and have shown large electromagnetic field enhancement [16]. To attest to the SERS efficiency of the HIF-Au NCs, the as-prepared HIF-Au NC_pH1.5_ was adopted as a colloid SERS substrate. Different concentrations of Raman probe, i.e., 2-MN, were added to the HIF-Au NC_pH1.5_ solution for the SERS measurements. As displayed in Figure 2d, several characteristic signals were observed in the SERS spectra. Typically, the peaks located at 1382.8 and 1619.2 cm^−1^ are attributed to the ring modes, the C−H bending mode is located at 1069 cm^−1^, and the peak at 769.3 cm^−1^ is dominated by the ring deformation. It was seen that the intensities of these peaks slowly decreased as the 2-MN concentration was reduced. Even when the 2-MN concentration was decreased to 10^−10^ M, their feature Raman peaks were observed, revealing the outstanding SERS property of the HIF-Au NCs. These results demonstrate the great potential of the synthesized HIF-Au NCs in SERS-sensing applications.

### 2.2. Evaluating the Electrochemical Oxidation Activity of the HIF-Au NCs

**Figure 4:**
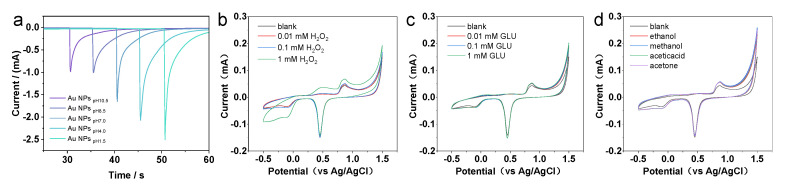
(**a**) I−T measurements. Evaluation of the electrochemical performance of the HIF-Au NC_pH1.5_-modified electrode under various mediums (**b**) in the H_2_O_2_ solutions, (**c**) in the Glu solutions, and (**d**) in different organic mediums.

As compared with Au NCs that are enclosed by a low catalytically active {111}/{100} facet, the presence of a highly reactive Au {311} facet in the HIF-Au NCs proved crucial for improving their electrochemical oxidation activity to target molecules. Moreover, as the weak adsorbed residual N_2_H_4_ species on the Au NCs can be easily removed or substituted, the surface of the synthesized HIF-Au NCs was rather clean. As shown in the i–t curves of the Au NCs prepared using N_2_H_4_ with different pH values, HIF-Au NCs have similar activity relationships to the CV curves (Figure 4a). Specifically, the Au NC_pH1.5_ and Au NC_pH4.0_ have similar activity relationships to the CV curves, which display more sensitive current changes than others. Although certain high-index crystal surface activity is observed at pH 7, it is markedly lower than that at pH 1.5 to 4.0. This may be due to the smaller particle size, which results in fewer high-index active sites. The Au NCs prepared under alkaline conditions showed poorer performance, indicating the crucial role of high-index crystal planes in improving detection sensitivity.

Subsequently, the electrochemical performance of the HIF-Au NC_pH1.5_-modified electrode was evaluated using H_2_O_2_, a commonly used electrocatalysis model molecule. The results indicate that in the supporting electrolyte (the Na_2_SO_4_ aqueous solution of 10–50 mM), the HIF-Au NC electrode was featureless in the range of −0.5–1.5 V, which is in accordance with the knowledge that Au is inert for the adsorption/desorption of H (at <0.3 V vs. Ag/AgCl) and the oxidation of surface Au atoms requires a high potential (>1.0 V). After the addition of H_2_O_2_ (Figure 4b), an evident oxidation peak was observed at −0.2V in the forward scan, indicating the reduction of H_2_O_2_ into O_2_. In addition, different concentrations of Glu and various organic solvents were also examined in this system (Figure 4c,d). It was revealed that none of them showed electrochemical oxidation, which demonstrates good selectivity of the newly developed HIF-Au NC electronic sensors.

**Figure 5:**
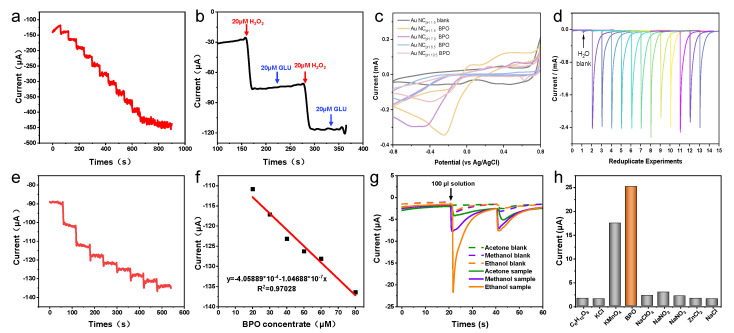
(**a**) Dose-dependence of the I−T measurements after adding increasing amounts of H_2_O_2_; (**b**) Changes in the current intensity after alternate addition of H_2_O_2_ and Glu. (**c**) Comparison of the CV result of various Au NCs for BPO; (**d**) Repeated experiments using the HIF-Au_pH1.5_. (**e**) Dose-dependence of the I−T measurements after adding increasing amounts of BPO and (**f**) their linear fitting result; (**g**) CV performed in various organic solvents; (**h**) Selectivity of the assay.

Furthermore, I−T measurements were performed to evaluate the sensitivity of the HIF-Au NC-based electrode for H_2_O_2_ at −0.2 V. With the gradual addition of the H_2_O_2_ solution from 20 to 300 μM, a rapid increase in the current intensity was observed (Figure 5a). The steady state current was achieved within a few seconds, confirming the rapid electrochemical response of the HIF-Au NCs to H_2_O_2_. The good linearity between the steady state current density and the H_2_O_2_ concentration supports the feasibility of the HIF-Au NC-based quantitative electrochemical analysis. As a common inference, the Glu aqueous solution (20 mM) caused no change in the current density, which was also negligibly influenced by the electrochemical response induced by the subsequent addition of H_2_O_2_ (Figure 5b).

### 2.3. Electrochemical Sensing Application of the HIF-Au NCs

We further tested the feasibility of employing the HIF-Au NCs as the electrochemical sensor in food safety applications. To this end, BPO, a highly concerning illegal food additive in flour, was selected as the target. Firstly, the electrochemical responses to various amounts of BPO in the aqueous phase were monitored. BPO was dissolved into ethanol, methanol, and acetone to mimic the corresponding extract. Twenty times higher current was observed in the presence of BPO, which implies that the HIF-Au NCs show high specificity for BPO, especially during the oxidative cleavage of the O–O bond, a process similar to that of H_2_O_2_ but more effective. The cyclic voltammetry curves of Au NCs were prepared using N_2_H_4_ with different pH values to compare the properties of different materials. Firstly, compared with the control group without BPO added, the cyclic voltammetry curves with BPO added showed significant reduction peak signals in the range of −0.2~−0.5 V (vs. Ag/AgCl). In addition, the reduction peak current intensity of Au NCs with lower pH was significantly higher than that of Au NCs prepared under alkaline conditions due to the higher sensitivity of high-index crystal planes to BPO. Therefore, Au NCs prepared under pH 1.5 are more suitable as the substrate for electrochemical sensors (Figure 5c). We conducted 12 repeated experiments using a substrate prepared at pH 1.5. Although there were fluctuations in the last six detection processes, stable detection performance was demonstrated in the first six repetitions, which is sufficient to complete the detection of conventional samples (Figure 5d).

As we expected, the HIF-Au NCs showed electro-oxidation inertness for organic mediums, which ensures their widespread use in food safety applications. Furthermore, we studied the electrochemical response of BPO extracted from the flour powder. CV performed in 10 mM ethanol, acetone, and methanol dissolved in a 50 mM Na_2_SO_4_ solution revealed that the onset potential for the oxidation of ethanol, acetone, and methanol was as high as −22.4 V (Figure 5g). These results confirm our hypothesis that the HIF-Au NC-based electrode showed a high electrochemical window for the detection of BPO.

The BPO-concentration-dependent current density curve is shown in Figure 5e, and their good linear-dependence responses are shown in Figure 5f. The corresponding current changes were correlated quantitatively with the BPO amount; a good linear relationship (R^2^ = 0.97) can be readily described using the linear equation: y = 4.06 × 10^−4^ – 1.05 × 10^−7^ C_BPO_ (C_BPO_ represents the concentration of BPO, uM). The detection limit for BPO was determined to be approximately 5.2 uM by including the control signal with three times the standard deviation. The high selectivity towards BPO was also demonstrated by examining its electrochemical response to various inferences. It was found that a 100 times higher concentration of inferences than BPO caused a negative effect on the results (Figure 5h).

Because BPO is often illegally added into flour as a bleaching agent, developing a sensitive assay in flour is of practical significance in the food safety field. To verify the practical applicability of this assay, commercial blank flour samples were spiked with BPO. The accuracy and precision of this method were verified, as demonstrated in Table 1. The results suggest the reliability of this method in real food samples. As demonstrated in Table 1, the detecting results suggest the reliability of this new method in real application. The results also indicate that by combining multiple advantages including simplicity, sensitivity, and accuracy, this assay has high application potential for the rapid screening of BPO.

## 3. Materials and Methods

### 3.1. Materials

Hydrogen peroxide (H_2_O_2_), disodium hydrogen phosphate (Na_2_HPO_4_), potassium dihydrogen phosphate (KH_2_PO_4_), potassium chloride (KCl), chloroauric acid (HAuCl_4_), hydrazine hydrate (N_2_H_4_·H_2_O), perchloric acid (HClO_4_), sodium chloride (NaCl), and glucose (Glu) were supplied by Sinopharm Chemical Reagent Co., Ltd. (Shanghai, China). 2-Mercaptonaphthalene (2-MN), isopropyl alcohol (IPA), and nafion (5%) were bought from Sigma-Aldrich Co., Ltd. (St. Louis, MO, USA). A 0.1 M Na_2_SO_4_ solution (pH 7.4) was used as the electrolyte in the electrochemical experiment. All reagents were used without further purification and all solutions were freshly prepared with ultra-pure water (18.2 MΩ/cm).

### 3.2. Synthesis of the HIF-Au NCs

A solution of HAuCl_4_ (1.0 mL, 50 mM) was added to ultra-pure water (50 mL) in a conical flask with magnetic stirring at 600 rpm. Subsequently, the N_2_H_4_ (500 μL, 0.1 M) aqueous solutions with pH adjusted from 1.5 to 10.5 were quickly added to the HAuCl_4_ solutions, respectively. The solution color changed from yellow to orange–red within 1 min. After continuously stirring for 10 min, the HIF-Au NC solutions were centrifuged at 3000 rpm for 5 min. The resulting HIF-Au NCs were collected as sediment and then redispersed in ultra-pure water for further use.

### 3.3. Characterizations

Scanning electron microscope (SEM) images of the HIF-Au NCs were obtained using a JEOL JSM-7900F (JEOL Ltd., Tokyo, Japan). Transmission electron microscope (TEM) images were observed using a JEOL JEM-2100F. High-angle angular dark-field scanning transmission electron microscope (HAADF-STEM) images were obtained on a Hitachi HF5000 (Hitachi, Tokyo, Japan). X-ray diffraction (XRD) patterns were recorded by a Bruker D8 Advance (Bruker, Bremen, Germany) using Cu Kα radiation (λ = 0.15406 nm). X-ray photoelectron spectroscopy (XPS, Axis Supra, Kratos, Manchester, UK) was used to evaluate the valence state of elements. Raman spectra were collected on an inVia Raman spectrometer (Renishaw, Wotton-under-Edge, UK) coupled with a confocal microscope.

### 3.4. SERS Measurement

The purified HIF-Au NCs were examined as a SERS substrate. After adding different concentrations of 2-MN dissolved into ethanol (20 μL), the HIF-Au NCs were transferred into a 96-well quartz plate with 1 cm optical length to collect the Raman spectra with 10-mW laser power, a 10× objective lens (NA = 0.25), and an integration time of 20 s.

### 3.5. Preparation of the HIF-Au NC-Modified Electrodes

Cyclic voltammetric (CV) and amperometric (IT) measurements were conducted on a CHI760 electrochemical workstation (Shanghai Chenhua Instrument Corporation, Shanghai, China) in a conventional three-electrode cell. The modified glassy carbon electrode (GCE), Ag/AgCl, and Pt wire were used as the working electrode, reference electrode, and counter electrode. A volume of 20 μL of the concentrated HIF-Au NCs was dropped onto the GCE surface and dried under ambient conditions. All solutions were purged with high purity N_2_ for at least 20 min to remove residual oxygen. The final packed cells were redispersed in 100 μL of PBS (pH = 7.4) for the real-time electrochemical experiments at an applied potential of −0.2 V.

### 3.6. Application of the Method in the BPO-Spiked Samples

In this study, a 0.1 M Na_2_SO_4_ solution was used as the electrolyte for the detection of BPO, with the CV potential selected to range from −1.0 V to 1.3 V at a scan rate of 100 mV/s. To prepare BPO-spiked food samples, a 10 mM BPO standard solution was added to 5.0 g of flour in a 50 mL centrifuge tube. BPO was extracted using various solvents including methanol, ethanol, or acetone, respectively, at 60 °C for 10 min, followed by centrifugation at 12,000 rpm for 5 min at 4 °C. The supernatant was then collected directly for further electrochemical detection.

## 4. Conclusions

In summary, HIF-Au NCs were synthesized via a facile one-pot wet-chemical method. In this aqueous-based reaction, the protonated N_2_H_4_ provided a moderate reduction kinetic for the formation of Au NCs. Benefitting from the rich 311 facets, the polyhedron Au NCs demonstrated enhanced electrocatalytic peroxide performance as well as outstanding SERS features. Using the HIF-Au NC-modified electrode pair, a simple, rapid assay for BPO was achieved with a limit of detection of 5.2 uM. The feasibility of this assay was further verified by reliably determining BPO in spiked flour samples. We hope this work will inspire more facile wet-chemical methods for NCs exposed with high-index facets to improve overall performance. Moreover, this study demonstrates the feasibility of improving the activity and selectivity of electrochemical analysis through exposed-crystal-face control and is expected to expand the application of electrochemical analysis into other fields.

## Figures and Tables

**Table 1:** Results for BPO detection in spiked flour samples (*n* = 3).

Sample	This Method		
	Spiked(uM)	Detected (uM)	Recovery (%)
Wheat flour	0	ND ^a^	-
	20.0	17.3	86.5
	50.0	46.7	93.4
Glutinous rice flour	0	ND ^a^	-
	20.0	18.1	90.5
	50.0	46.3	92.6

^a^ Not detected.

## Data Availability

The original contributions presented in the study are included in the article, further inquiries can be directed to the corresponding author.
